# Magnetic oxygen stored in quasi-1D form within BaAl_2_O_4_ lattice

**DOI:** 10.1038/s41598-019-51653-4

**Published:** 2019-10-22

**Authors:** Martina Vrankić, Ankica Šarić, Sanja Bosnar, Damir Pajić, Jure Dragović, Angela Altomare, Aurelia Falcicchio, Jasminka Popović, Marijana Jurić, Mladen Petravić, Ivana Jelovica Badovinac, Goran Dražić

**Affiliations:** 1 Division of Materials Physics, Center of Excellence for Advanced Materials and Sensing Devices, Ruđer Bošković Institute, Bijenička 54, 10000 Zagreb, Croatia; 2 Division of Materials Chemistry, Ruđer Bošković Institute, Bijenička 54, 10000 Zagreb, Croatia; 3 Department of Physics, Faculty of Science, University of Zagreb, Bijenička 32, 10000 Zagreb, Croatia; 4 Institute of Crystallography-CNR, via Amendola 122/o, 70126 Bari, Italy; 5 Department of Physics and Center for Micro- and Nanosciences and Technologies, University of Rijeka, Radmile Matejčić 2, 51000 Rijeka, Croatia; 6 Department of Materials Chemistry, National Institute of Chemistry, Hajdrihova 19, Ljubljana, Slovenia

**Keywords:** Materials science, Magnetic properties and materials, Physics

## Abstract

Inorganic materials that enable a link between the storage and release of molecular oxygen offer a fertile ground in continuous quest for the applications that can potentially reduce energy consumption and thus minimize adverse effects on the environment. Herein, we address reversible intake/release of an oxygen within the BaAl_2_O_4_ material as evidenced by unexpected magnetic ordering. Magnetic measurements unveil that an oxygen is stored in the form of condensed matter, creating a kind of low dimensional, chain-like assembly within the tunnels of BaAl_2_O_4_ structure. We demonstrate that oxygen is adsorbed simply by staying in air, at ambient conditions, and released relatively quickly by staying in the He or other gas atmosphere of several millibars pressure even at 300 K.

## Introduction

There is an ever-growing demand for the compounds showing exceptional and unique properties that can be used in the energy conversion and saving processes. Thus, according to previous research activities, a special emphasis is placed on highlighting novel materials which will be able to satisfy high demands of modern, energy-related technologies^1–3^ having at same time a very strong tendency towards economic and environmental aspects of the production and application. Generally, the materials that are able to bind and store oxygen are major factor in many novel applications that help to reduce emissions, lower energy consumption and minimize environmental impact. In recent years a number of oxygen-storage material families have been discovered. The most extensively investigated oxygen storage families are based on the compounds such as ceria^4–7^, perovskites^8–10^, rare-earth manganese oxides^11,12^, delafossites^13,14^, LuFe_2_O^15,16^ and YBaCo_4_O_7_^17–21^. Namely, the most important aspects of an oxygen non-stoichiometric YBaCo_4_O_7+δ_ based materials were extensively reviewed in several publications reported by the Karppinen group^17–21^ showing that the oxygen-storage capacity of layered cobalt oxide compounds is better than those of conventional oxygen-storage materials as reversible oxygen absorption/desorption process occurs around 300 °C. However, the disadvantage of this system is poor thermal stability as it decomposes at relatively low temperature, just above 600 °C, under an oxygen-containing atmosphere^17^. Still, the phase stability of the YBaCo_4_O_7+δ_ can be enhanced through a chemical substitution involving any of three cation constituents^21^. Recently, Hervieu *et al*.^15^ reported an oxygen storage ability of ferroelectric LuFe_2_O_4+x_ (0 < x < 0.5) and its cycling possibility coupled to transport and magnetic properties. This ferrite material is notable example of an oxygen storage material, as an oxygen uptake starts at low temperature, around 200 °C. Moreover, the reversibility of the reaction was also confirmed by the evolution of magnetic properties^16^.

Generally, there are several major demands which have to be met when dealing with possible candidates showing an oxygen storage ability; material should have a high oxygen storage capacity (OSC), a sufficiently low operation temperature and adequate thermal stability^22^. Typically, all materials showing an oxygen storage behaviour are characterized by the oxygen non-stoichiometry which allows continuous cycling between reduced and oxidized form usually associated with an existence of reversible structural transformation or with the emptying and filling of the oxygen or cation vacancies.

In our quest for new types of materials that could bind and store oxygen, the barium aluminate (BaAl_2_O_4_) emerged as a potential candidate owing to its structural features, exceptional high thermal stability (1815 °C), non-toxicity and low price. Barium aluminate crystallizes in hexagonal crystal system within the trydimite-like structure type^23^. It has to be stressed that, neither barium nor aluminium belong to the group of transition metals, there are no multiple oxidation states, thus an oxygen non-stoichiometry typical for the oxygen storage families, is not expected in the case of the barium aluminate. Generally, barium aluminate and its derivatives have proven to be useful in various technologies such as the production of modern fluorescent lamps, cathode ray tubes, field emission displays (FEDs), plasma display panels (PDPs) and fibre amplifiers^24,25^. The BaAl_2_O_4_ doped with transition metal and/or rare earth ions displays a long afterglow luminescence^26,27^. Besides, ferroelectric^23^, catalytic^28^, and advantageous hydraulic hardening^29^ properties are also characteristic for the BaAl_2_O_4_ compound. According to the literature, one might notice that a considerable effort has been made to elucidate optical performance of this compound^30–32^. On the contrary, there is a relatively small number of the studies revealing significant contribution of the barium aluminate in the field of the energy conservation and positive environmental impact. Accordingly, Casapu *et al*. reported systematic investigation of the barium aluminate formation and decomposition processes, which can greatly affect the NO_x_ storage-regeneration activity^33^, while in the works of Hodjati *et al*. a good potential of the BaAl_2_O_4_ for NO_2_ trapping^34,35^ has been demonstrated. However, possible new aspect of the barium aluminate application in the field of an oxygen storage materials has not been proposed in the literature up to date.

In this paper we report on the investigation of a reversible oxygen intake process in BaAl_2_O_4_ based on its unusual and surely quite unexpected magnetic behaviour, despite both the Al and Ba cations being typical non-magnetic species.

## Results

### X-ray photoelectron spectroscopy

**Figure 1:**
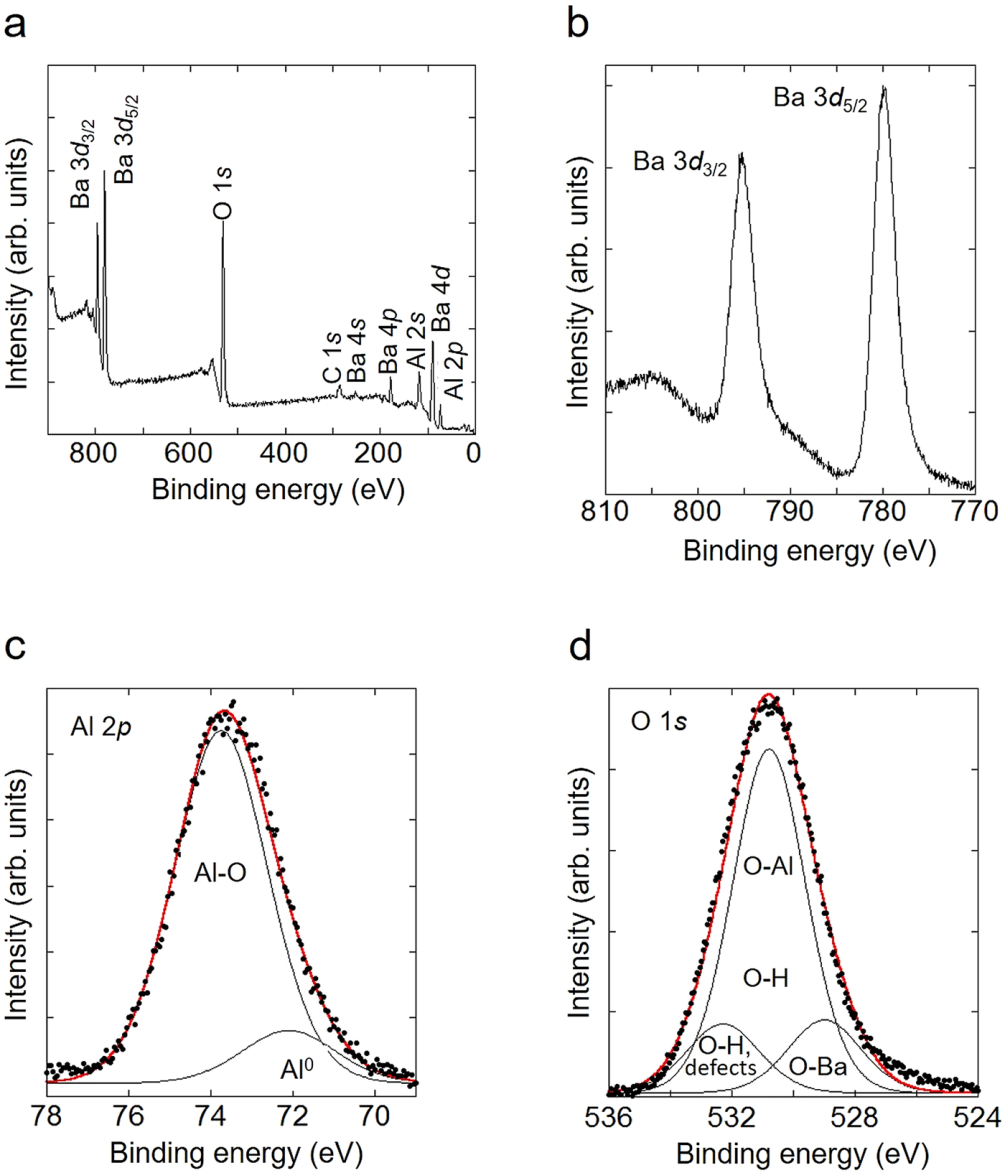
XPS characterization. (**a**) Survey XPS spectrum taken from the surface of a BaAl_2_O_4_ sample. (**b**) Ba 3*d* core-level photoemission spectrum from a BaAl_2_O_4_ surface. (**c**) The deconvoluted Al 2*p* spectrum from a BaAl_2_O_4_ sample. (**d**) The O 1*s* spectrum of a BaAl_2_O_4_ sample surface, fitted with three components. Closed circles represent experimental XPS data and solid lines convolution of Gaussians and Lorentzians.

The BaAl_2_O_4_ surface was characterized by the XPS around the Ba 3*d*, Al 2*p* and O 1*s* core levels, while broad survey spectrum was also taken to confirm low level of the impurities present on the surface. Indeed, survey spectrum in Fig. 1a does not show any impurity-related peaks except for typical carbon C 1*s* peak from a carbon contamination. This undoubtedly confirms the chemical composition of the titled sample, and also, which is exceptionally important in this case, proves the absence of possible impurities that might eventually be a cause of artifact(s). On the other hand, photoemission peaks from all matrix elements are present in the spectrum, indicating well defined and clean surface.

The Ba 3*d* photoemission region, shown in Fig. 1b, is characterized by well separated spin-orbit components, Ba 3*d*_5/2_ and Ba 3*d*_3/2_ at binding energies (BE) of 780 and 795.3 eV, respectively, in full agreement with the literature data^31,36^. The photoemission peaks around the Al 2*p* (Fig. 1c) and O 1*s* (Fig. 1d) core levels exhibit large FWHM of 2.8 eV and 2.9 eV, respectively, indicating several spectral components. Therefore, we fitted both spectra with several Gaussian–Lorentzian functions. The good fitting of the Al 2*p* spectrum in Fig. 1c is possible only by introducing two fitting components at the BE of 72.1 and 73.8 eV. We assign these components to metallic Al (Al^0^) and Al–O bonds, respectively. Also, it can be noted that the 2*p* XPS spectra usually exhibit the spin-orbit splitting to the 2*p*_3/2_ and 2*p*_1/2_ states but in the case of the Al 2*p*, this splitting is too small (0.4 eV) to be resolved. The O 1*s* spectrum was deconvoluted into three Gaussian–Lorentzian components originating from the O–Ba bonds at the BE of 529 eV and O–Al bonds at 530.8 eV. The third peak at the BE of 532.3 eV is assigned to the O–H groups, chemisorbed oxygen or even the oxygen defect states^37^ found on many surfaces. This assignment supports the results of the FTIR spectroscopy (Supplementary Fig. 1); the broad band centred around 3480 cm^−1^, and one located at 1640 cm^−1^ originate from the stretching mode of hydroxyl group and the deformation vibration of H–O–H typical for the water molecules absorbed by the sample, respectively^38,39^. The stretching band at ~1435 cm^−1^ is assigned to carbon, that exists as a carbonate in BaAl_2_O_4_ sample^31,40,41^. The adsorbed CO_2_ could be driven off by heating the BaAl_2_O_4_ sample overnight at high temperature (Supplementary Fig. 2). Absorbed peaks centred at 430, 630 and 830 cm^−1^ are typical for the barium aluminate species^42^.

### Structural characterization

The room temperature (RT) X-ray powder diffraction (XRPD) pattern collected after the calcination of BaAl_2_O_4_ precursor revealed that completely single phase product was obtained. The data reduction for the BaAl_2_O_4_ sample was performed using the EXPO2014 program following the standard steps^43^. Indexing of the powder pattern using the N-TREOR09 led to plausible hexagonal unit cell with initial parameters *a* = *b* = 5.2196 Å and *c* = 8.7879 Å having a total volume of ~207.2 Å^3^. The space group was determined as non-centrosymmetric *P*6_3_22 and the number of formula units per unit cell was calculated to be Z = 2 from the packing considerations.

**Figure 2:**
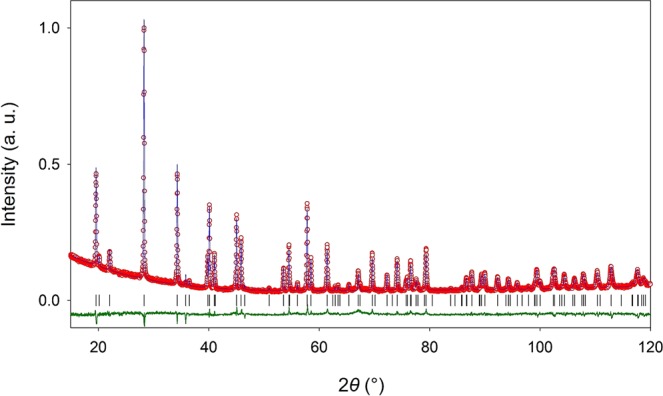
Structural characterization. Final observed (red circles) and calculated (blue solid line) powder diffraction profile for the BaAl_2_O_4_ sample at ambient temperature. The lower green solid line shows the difference profile and black tick marks show reflection positions.

The structure reinvestigation showed that BaAl_2_O_4_ at RT crystallizes in hexagonal *P*6_3_22 space group instead in a *P*6_3_ group as previously reported by Huang *et al*.^24^. In final step, obtained structural model was refined by the Rietveld method with excellent agreement between observed and calculated profiles achieved for the titled compound (*a* = *b* = 5.22111(6) Å, *c* = 8.78981(14) Å) at RT (agreement factors, *R*_wp_ = 5.94%, *R*_p_ = 4.45%) as shown in Fig. 2. The Rietveld refinement gave the value for the Ba1–O1 bond length of 2.902(4) Å and the Ba1–O2 bond length of 3.0144(4) Å. In the structural AlO_4_ tetrahedra the Al1–O1 bond lengths were 1.737(6) Å and the Al1–O2 1.713(6) Å. The refinement procedure involved refinement of background parameters, diffraction-line profile parameters, lattice parameters *a* and *c*, atomic position parameters, and temperature factors for all present atoms. Isotropic vibration modes were assumed for all atoms. Refinements of the cation occupancies, as well as O1 and O2 site occupancies showed that all sites are full and thus indicating no sign of vacancy formation. The final structural and microstructural parameters are summarized in Supplementary Table 1, along with reliability factors confirming a validity of the refinement.

### Microstructural characterization

**Figure 3:**
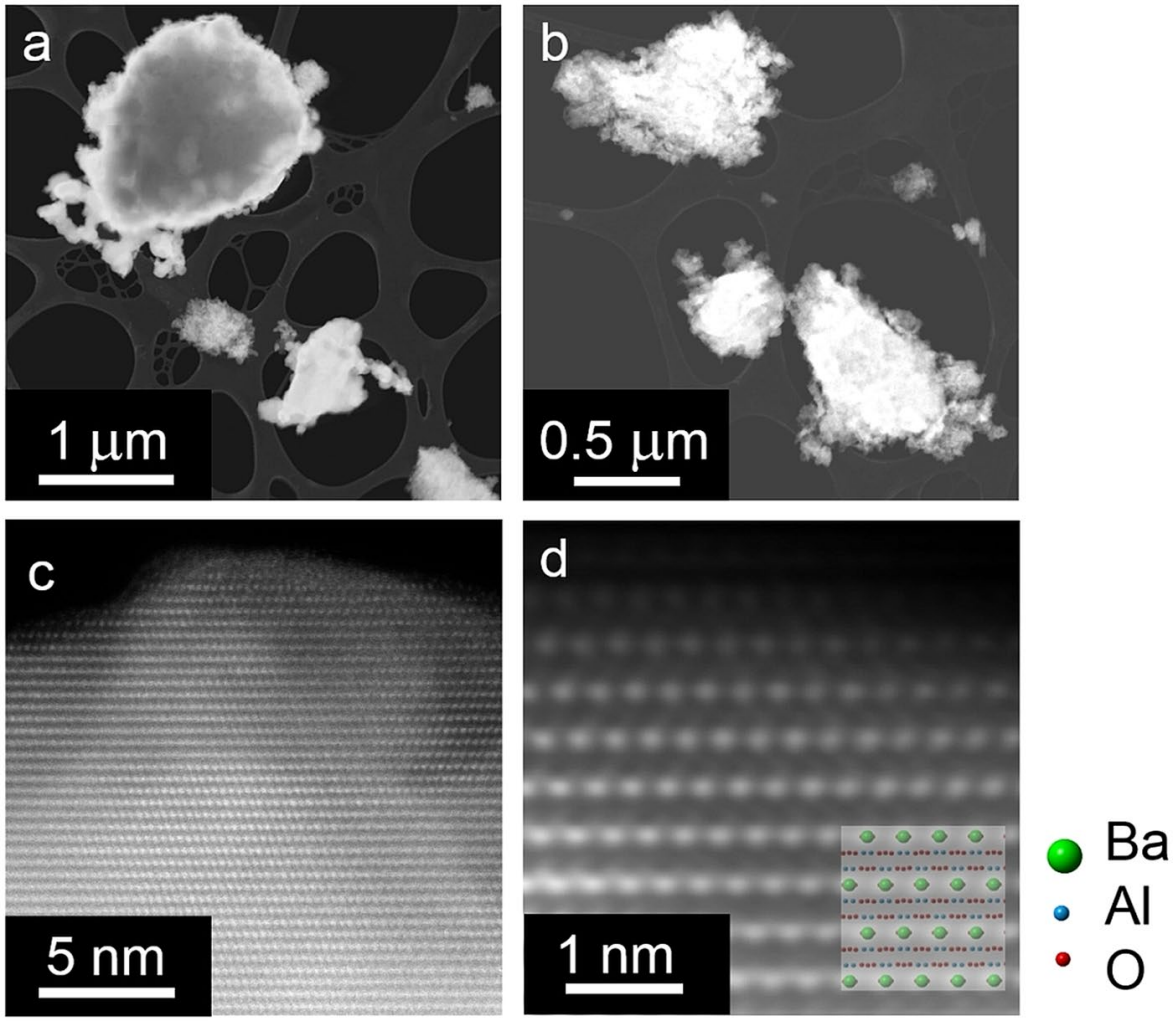
Microstructural characterization. (**a**,**b**) The HAADF-STEM images of a BaAl_2_O_4_ sample. Agglomerates of the material of the size up to 1 μm are dispersed on a lacey-carbon coated Cu grid. (**c**) Atomic resolution image [021] zone axis showing the positions of Ba atom columns. (**d**) Enlarged image showing that superimposed hexagonal P6_3_22 BaAl_2_O_4_ structure model nicely fits to the structure.

Furthermore, the scanning transmission electron microscopy (STEM) revealed that the particles comprising the powder material were irregularly shaped and assembled into the agglomerates of size up to 1 μm (Fig. 3a,b). The position of Ba atoms, which are practically only atom columns that could be seen in the HAADF-STEM (High Angle Annular Dark-Field STEM) images due to high atomic number, fit very well to hexagonal *P*6_3_22 structural model (Fig. 3c,d). From atomically resolved HAADF-STEM images we concluded that no substantial amount of the crystal defects were present in the material.

### Magnetization study

**Figure 4:**
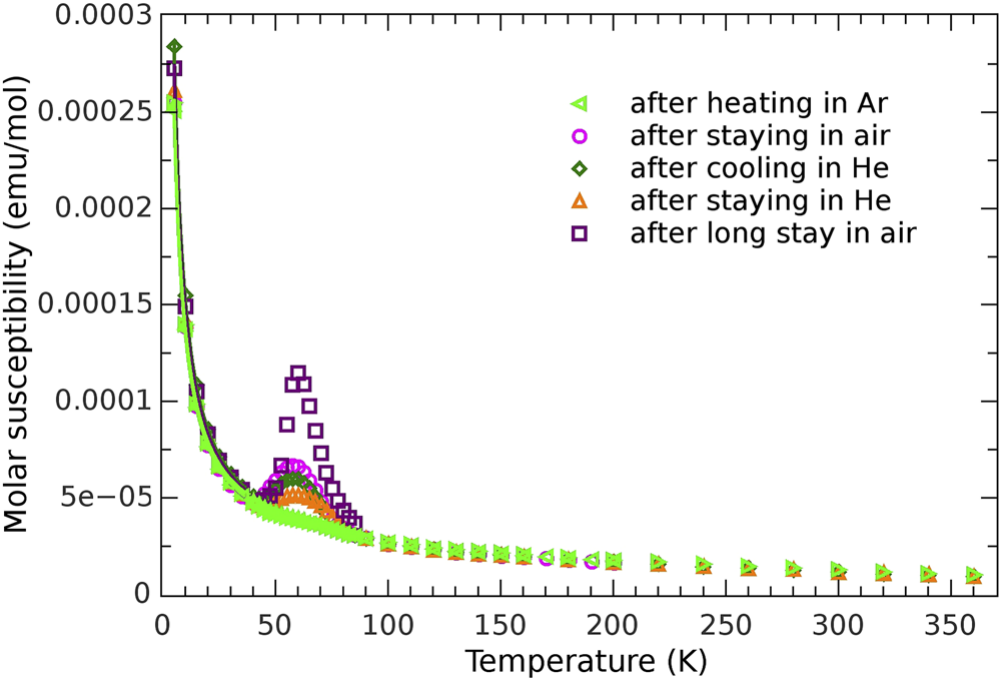
Magnetic susceptibility and change of the oxygen peak. Temperature dependence of the molar susceptibility measured in the magnetic field of 0.1 T. Black circles represent the measurement of the as-prepared sample inserted directly to 5 K temperature. Red squares show typical dependence obtained after staying of the sample at the room temperature and atmosphere. Measurement performed after heating to 360 K in helium atmosphere of several millibars shows disappearance of oxygen peak from the susceptibility curve measured after that, as denoted with green triangles. Blue rhombs show the measured curve in which oxygen peak returned after staying of the sample in air under ambient conditions. The solid lines are fitting curves.

The temperature dependence of the magnetization *M*(*T*) for an as-prepared polycrystalline sample in a form of pressed pellet was measured in different magnetic fields, and the susceptibility curves for 0.1 T are shown in Fig. 4.

Prior to magnetic measurements, possible influence of a pressure on the structure and/or composition of the sample was checked; no difference between the pellet and original powder was observed. It is also worth of mentioning, that there is no doubtful oxygen contamination inside the magnetometer, as we checked it prior and after the measurements of BaAl_2_O_4_ with other small signal samples of similar compounds which did not show any magnetic anomaly around 50 K.

The first magnetic measurement was performed after quick insertion of the pellet down to liquid He temperature. Magnetization curve shows three distinctive temperature regions. Below 40 K the *M(T)* shows a paramagnetic-like behaviour although there are no magnetic ions present in the BaAl_2_O_4_ compound. From the Curie fit to measured data (*χ* = *C/T*), obtained Curie constant *C* gives an amount *p* = (0.304 ± 0.005)% of entities of spin 1/2 per formula unit. Together with the XRPD and XPS results it confirms, even more accurately, that there are no paramagnetic impurities present in samples in considerable amount.

Many datasets, collected below 35 K, confirmed, undoubtedly, the constant amount of paramagnetic entities present after different thermal treatments. The introduction of Weiss parameter *θ* into the fit contributes only slightly to its quality, with the *θ* values between −0.06 K and −0.6 K with large relative errors, showing that (anti)ferromagnetic interactions between those paramagnetic entities have no significance, as one can expect for this system. The observed paramagnetic term could originate from unpaired electrons sitting around the defects, such as vacancies. However, none of experimental techniques employed in our study point out to the presence of any vacancies within the aluminate lattice as well as other detectable paramagnetic impurities, so that the origin of this upturn can not be fully explained. At the temperatures above 100 K magnetic signal was very small and immerged in noise, precluding any quantitative analysis. Still, this poor signal clearly indicates negligibly small presence of paramagnetic centres. The χ*(T)* measurements in Fig. 4 show the most intriguing behaviour between 45 and 75 K that points out to possible role of the oxygen in magnetization measurements. Namely, it is well known that the oxygen has antiferromagnetic^44^ transition at 50 K. Therefore, it is expected to observe a small peak in the *M*(*T*) measurements due to this transition, usually coming from small amount of an air enclosed within measuring ampoule often used in the magnetometers. However, in our experimental set-up we did not use the ampoule. Instead, we fixed the pellet within open straw, evacuated whole assembly and flushed it with the He several times before inserting it to low temperature region. Thus, no oxygen contamination is expected around the sample and we assign the measured bump in the *M(T)* in Fig. 4 to the sample magnetization. Another interesting point is related to very special shape of the maximum; the bump has no sharp peak characteristic for the oxygen antiferromagnetic^44^ transition. Instead, it is smeared broadly over a large temperature interval and has very smooth and wide maximum. As we will argue in the discussion section, this kind of the broadening is characteristic for low dimensional antiferromagnetism^45–47^, rather than a 3D antiferromagnetic order. Namely, a very interesting phenomenon is diminishing, or even complete disappearance of magnetization maximum after moderate heating. The first set of measurements, performed from 5 K to 200 K (after fast cooling from ambient conditions at 300 K down to 5 K in less than a minute by direct insertion of the sample rod in the cryostat) showed pronounced and well resolved maximum. After that, the sample was cooled again as fast as possible (10 K/min) from 200 K down to 5 K and then measured during the heating from 5 K up to 300 K. The maximum was still well pronounced with only slightly lower intensity than in previous run. In next step, the sample was heated to 360 K and stabilized there for 1 hour in fixed atmosphere of several millibars of the He gas (standard condition for this type of the magnetometer).

After this procedure, the maximum on the *M*(*T*) curve vanished (almost disappeared) and did not re-appear in any consecutive *M*(*T*) measurement, as long as the sample remained within the magnetometer (Fig. 4). The magnetization behaviour changed again after the sample was taken out of the magnetometer and left at RT in an air for some time (one day or even several months). The consecutive measurements showed same *M(T)* behaviour as before: first of all, a very well pronounced maximum around 55 K was visible again, but, as before, it diminished with the cycling of temperature up and down, and ultimately, disappeared completely after leaving the sample, for a while, above RT within the He atmosphere (Fig. 4). The above phenomenon was entirely reproducible confirming quite clearly that after leaving the sample at the room ambient conditions the sample’s magnetization shows an oxygen-like maximum in the *χ*(*T*) curve, that diminishes after cycling the temperature in the atmosphere of several millibars of the He, and finally disappears completely after heating the sample to 360 K within the magnetometer. Such behaviour strongly confirms that the air/oxygen is driven out of polycrystalline sample if it stays at the temperature slightly higher than RT while exposed to the He atmosphere at moderate vacuum (i.e., air is exchanged with the He). On the other hand, the air/oxygen occupies relatively quickly the interior of evacuated sample left in an air at ambient temperature. The latter is supported by the thermogravimetric (TG) measurements which complement a tendency of BaAl_2_O_4_ to intake the oxygen in the wide temperature range, from RT up to 600 °C (Supplementary Fig. 3).

Further study of the oxygen-related magnetization was performed on same pellet exposed to the room conditions for a long time. This pellet was heated at 400 K in the Ar atmosphere within quartz tube, evacuated and flushed with the Ar alternately several times in order to exclude any presence of oxygen. After heating for 1 hour, the pellet was quickly transferred into the magnetometer (in less than 2 minutes), rapidly cooled down to 5 K followed immediately by magnetization measurements.

**Figure 5:**
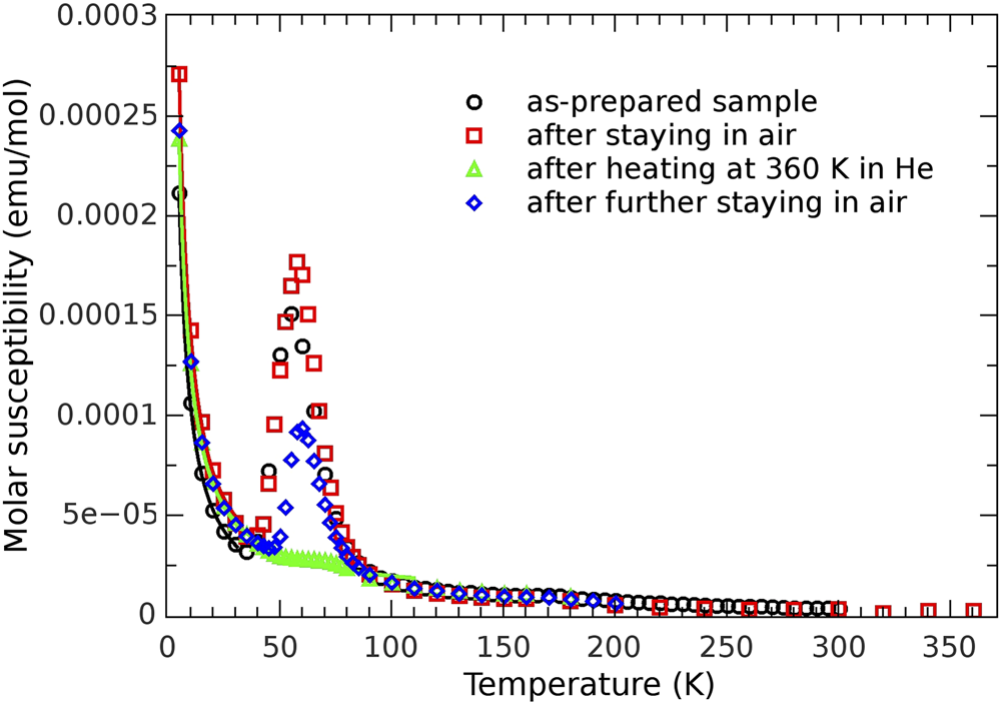
Magnetic susceptibility after thermo-atmospheric treatments. Temperature dependence of the molar susceptibility measured after different thermo-atmospheric treatments. First, the sample was heated to 400 K and stayed in pure argon atmosphere for 1 hour and then quickly inserted into the magnetometer to 5 K temperature, after that the susceptibility curve represented with green triangles showed no oxygen peak. Next, the sample was left on room conditions in air, after that the oxygen peak is again present during magnetic measurement (purple circles). This peak diminishes in measurements performed after short stay in magnetometer in helium atmosphere of several millibars followed by slow cool-down (dark-green rhombs) and further diminishes after an even longer stay in magnetometer in helium atmosphere (orange triangles). In measurement performed after a long stay in air at room conditions, the oxygen peak becomes very pronounced again (dark squares). The solid lines are fitting curves.

No maximum in *χ*(*T*) was observed, as shown in Fig. 5, proving, undoubtedly, that the adsorbed oxygen was, indeed, the source of the observed maxima in previous *χ*(*T*) curves from Fig. 4.

**Figure 6:**
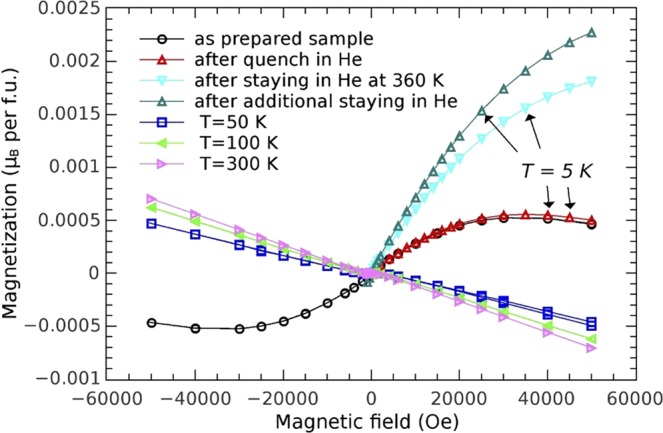
Isothermal magnetic curves after different thermo-atmospheric treatments. The field dependences of magnetization measured at temperatures 300 K, 100 K and 50 K show the diamagnetic behaviour of the compound. Several isothermal magnetization curves marked with arrows are measured at 5 K temperature after different thermo-atmospheric treatments and paramagnetic contribution in several steps becomes more and more pronounced as the oxygen is expelled and its contribution is suppressed.

Moreover, if the transfer from furnace tube to the magnetometer takes about 10 minutes, the maximum in the χ(*T*) measurements reappears, although with a slightly lower intensity than in the Fig. 6a, revealing that several minutes of an air exposure at ambient conditions represents characteristic time scale for the Ar-air interchange process. The above thermal procedure in the Ar atmosphere was repeated several times for reproducibility on all pelleted samples. Consequently, all samples showed same behaviour of the *χ(T)* each time.

Further demonstration of antiferromagnetic contribution to the magnetization can be obtained from the field dependence of the magnetization, *M*(*H*), measured at different temperatures and after different thermo-atmospheric treatments, as shown in Fig. 6. At higher temperatures, the *M*(*H*) curves exhibit linear dependence pointing to diamagnetic state of the compound. More information is obtained from low temperature *M*(*H*) measurements. As an example, we show in Fig. 6 four characteristic curves measured at 5 K. Their curvature is clear fingerprint of paramagnetic contributions, in full agreement with the *χ*(*T*) analysis. However, observed large differences between them originate primarily from the oxygen content: a large contribution from the oxygen in as prepared or quenched samples diminishes the magnetization, while, on the contrary, reduced amount of the oxygen in samples exposed to the He atmosphere lowers diamagnetic contribution and, therefore, increases the value of the *M*(*H*) curves.

## Discussion

**Figure 7:**
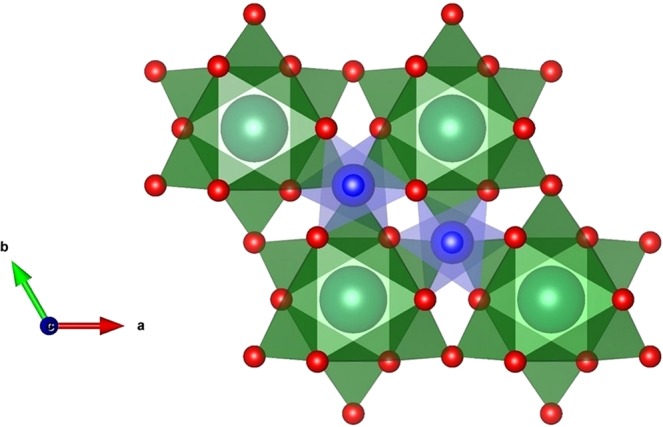
Crystal structure of barium aluminate. The stuffed–trydimite structure of barium aluminate at RT (space group *P*6_3_22, lattice constants *a* = *b* = 5.2211(1) Å, *c* = 8.7898(1) Å and V = 207.501(5) Å^3^). The unit cell is depicted by solid lines. Green and blue polyhedra highlight BaO_9_ and AlO_4_ units, respectively, and red spheres represent O. A three-dimensional network of the corner-sharing AlO_4_ tetrahedra, has tunnels along [001] direction where the relatively large, compensating Ba^2+^ ions are located. The existence of such passageways might provide potential transportation of oxygen within a crystal lattice.

Detailed analysis including the XPS, XRPD, STEM, TGA and magnetization has revealed good opportunity for the barium aluminate in the gas storage application. Unlike other metal oxide materials reported in the literature, oxygen storage ability of the BaAl_2_O_4_ originates neither from structural changes (i.e., phase transitions) nor from the changes of the oxidation states of metal cations which might lead to the oxygen non-stoichiometry. In the case of BaAl_2_O_4_, it seems that the mechanism of reversible process of the oxygen storage and release as demonstrated by unexpected and unusual magnetic behaviour, might be related to some specific structural features of stuffed trydimite-like structure. Structural determination from the XRPD data showed that the BaAl_2_O_4_ lattice contains tunnels along the *c*-direction (Fig. 7) which allow uptake and storage of oxygen.

Additionally, it has been recently reported that the BaAl_2_O_4_ structure can be, in fact, considered as a quite flexible one, as demonstrated by the phonon mode calculations associated with considerable tilting of the AlO_4_ tetrahedra^48^.

**Figure 8:**
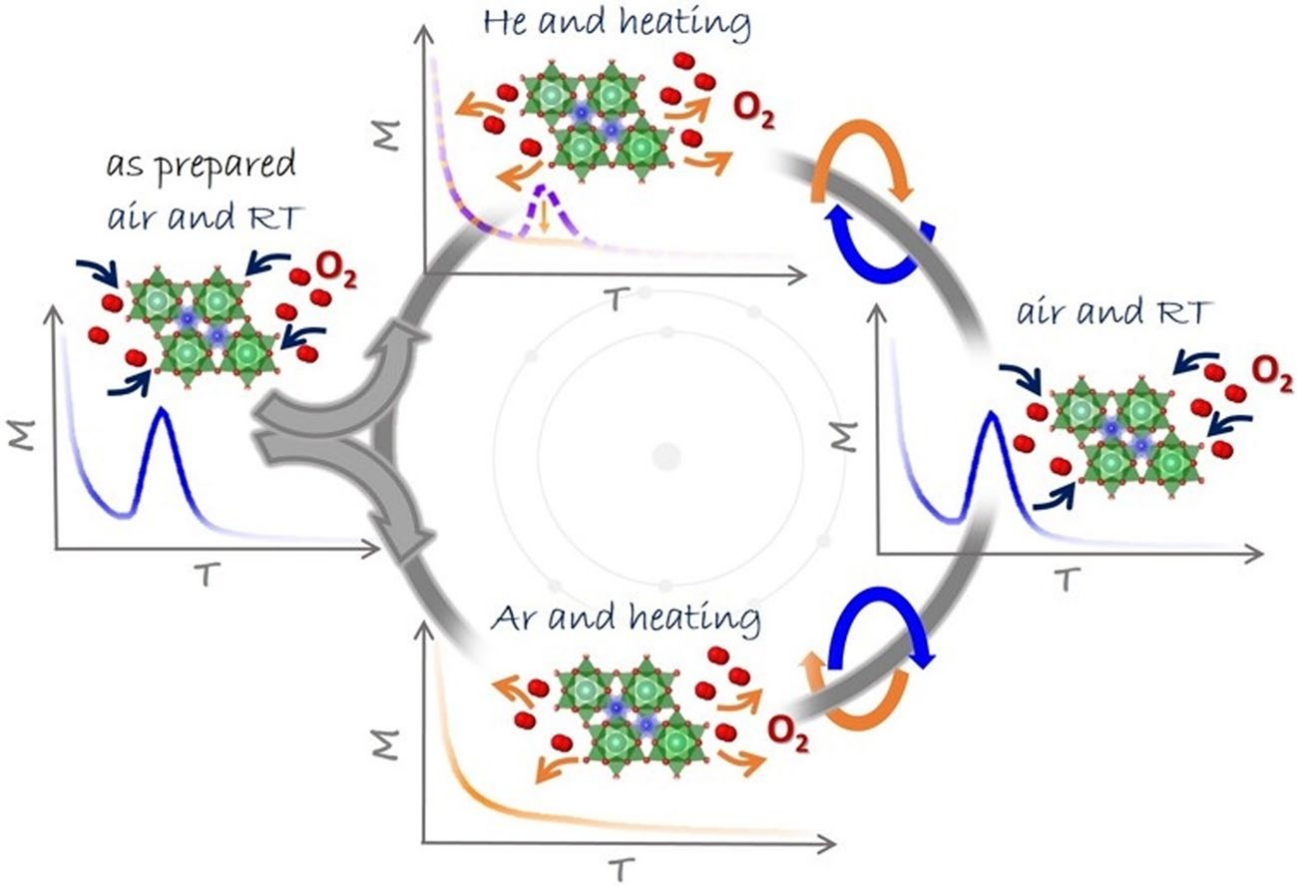
Influence of thermo-atmospheric treatment on the magnetization serving as a detection of the oxygen. Original sample shows presence of the oxygen. Either heating in He atmosphere within the magnetometer, or heating in Ar atmosphere within previously evacuated tube furnace, leads to the disappearance of an oxygen peak in magnetic measurement. The same sample attains the oxygen peak again after staying in air. The processes of intake and expelling of oxygen are completely recyclable, as confirmed from magnetic measurement.

Considering all experimental results, schematically illustrated in Fig. 8, following interpretation of the results is proposed: the presence of tunnels within the aluminate lattice allows oxygen to enter into interior space where it condenses. At low temperatures, oxygen is stored in the form of condensed matter agglomerates, as confirmed by the presence of maxima in magnetic susceptibility, *χ*(*T*), curves.

Nevertheless, detailed inspection of these maxima reveals a wide and smooth shape, quite different from typical antiferromagnetic maxima which would result from the 3D ordering of crystalline oxygen^44^. This observation confirms that oxygen, in fact, forms the 1D chain-like assembly within the tunnels of the BaAl_2_O_4_, constrained in thickness down to a very small number of atoms. It is not possible to treat this assembly as a spin chain using the Bonner-Fisher model for ideal spin chains^49^ due to many possible irregularities and inhomogeneities of the oxygen stacking in produced material.

Besides this quasi-1D form of storage, intrinsic to crystal structure of BaAl_2_O_4_, the grain boundaries of polycrystalline samples could contain condensed 2D form of oxygen, which is also expected to contribute to the magnetization with a wide maximum at similar temperature. The size and shape of the oxygen 1D and 2D crystalline condensates may influence the peak position of the *χ*(*T*) curve, while the superposition of wide distributions of these forms results in a very broad maximum of the *χ*(*T*). As the sample is heated up, slightly above RT, either in the He atmosphere within the magnetometer or in the tube furnace with the Ar atmosphere, oxygen is released and escapes from the interior of the aluminate lattice.

Additionally, present study shows that oxygen is adsorbed relatively quickly even at ambient conditions not only when fresh batches were used, but also with samples containing the Ar or He. Also, stored oxygen can be driven relatively quickly out of the samples exposed to the atmosphere of several millibars pressure of the He or other gases. This process of exchange of the gases can be repeated in any order and is recyclable, giving always the signature in magnetic susceptibility peak when the oxygen is present.

In summary, magnetic measurements, showing unexpected results at the first glance, revealed that oxygen can be stored and released from the BaAl_2_O_4_ polycrystalline samples exposed to mild conditions (i.e., temperatures around RT, with weak under-pressure, and for relatively short time). It is important to stress here that stable structure of the BaAl_2_O_4_ polycrystals was preserved during all these processes, despite a large stress developed upon the oxygen/air crystallization. The same storage mechanism in the BaAl_2_O_4_ polycrystalline samples should be also operational for different selected gases. However, in present study it has been investigated only for oxygen due to unique antiferromagnetic transition in crystalline oxygen, not existing in other gases.

## Methods

### Materials

All the reagents were used as purchased with analytical grade without any further purification. High purity aluminium nitrate nonahydrate Al(NO_3_)_3_·9H_2_O (Fisher Chemical, USA), was used as Al, and a barium nitrate, Ba(NO_3_)_2_ (Fisher Chemical, USA) as Ba precursor.

### Preparation of BaAl_2_O_4_ powder

Powder samples of the BaAl_2_O_4_ were prepared by hydrothermal method with a post-annealing treatment. Aqueous solutions of the aluminium nitrate nonahydrate and barium nitrate were prepared by dissolving stoichiometric amounts of salts in a Milli-Q water. The aqueous solutions were mixed in molar ratio and additionally homogenised by mixing and adding the 2-hydroxypropane-1,2,3-tricarboxylic acid, C_6_H_8_O_7_·H_2_O. Final solutions were prepared by adding the excess of 25% NH_3_ in H_2_O. Each of prepared solutions was subjected to the precipitation in an autoclave at 170 °C during 24 h, under static condition. Obtained precipitates were separated from a supernatant by using a centrifuge, additionally washed off with the Milli-Q water and dried at 60 °C. After that the powder was heated up to 1100 °C in the furnace with static air at a heating rate of 10 °C/min, and calcined at that temperature for 4 h. The thermal treatment generated a sample of light grey colour.

### IR Characterization

The infrared spectrum was recorded in the 4000–350 cm^−1^ region using sample in a form of the KBr pellet with a Bruker Alpha FTIR spectrometer.

### TGA measurements

Thermal stability characteristics and oxygen intake properties were evaluated by means of TG experiment carried out upon heating in controlled atmosphere (i.e., pure O_2_). A thermogravimetric analysis (TGA) was performed on a Mettler–Toledo TGA/SDTA851^e^ thermobalance using an alumina crucibles under pure oxygen stream with the heating rate of 2 °C min^−1^, from RT to 600 °C.

### XPS study

The X-ray photoelectron spectroscopy (XPS) spectra were recorded using the SPECS laboratory system equipped with a Phoibos MCD 100 electron analyser and monochromatic source of the Al Kα X-rays of 1486.74 eV. A typical pressure in the UHV chamber during the XPS analysis was in the 10^−7^ Pa range. For the electron pass energy of hemispherical electron energy analyser of 10 eV used in present study, overall energy resolution was around 0.8 eV. The experimental photoemission spectra were analysed by the Unifit software^50^ and simulated with several sets of mixed Gaussian-Lorentzian (G-L) functions (with the product version and mixing ratio of the G to L of 70% to 30%) with the Shirley background subtraction.

### Structural characterization

The RT X-ray powder diffraction (XRPD) patterns were collected by using automated Rigaku RINT2500 laboratory diffractometer (50 kV, 200 mA) equipped with a Si strip Rigaku D/teX Ultra detector. Asymmetric Johansson Ge crystal was used to select monochromatic Cu Kα1 radiation (λ = 1.54056 Å). The angular range 15–120° (2*θ*) was scanned with a step size of 0.02° (2 *θ*) and counting time of 1.0 s/step. Measurements were carried out in transmission mode, by introducing the sample in a special glass capillary (outer diameter = 0.3 mm and capillary thickness = 0.01 mm) and mounted on the axis of the diffractometer. In order to reduce the effect of possible preferential orientation, the capillary was rotated during measurements to improve the randomization of orientations of individual crystallites.

All the steps of the ab-initio structure solution process (i.e., indexation procedure by N-TREOR09, space-group determination, integrated intensity estimation, crystal structure solution by Direct Methods and structure model optimisation) were performed using the EXPO software^43^. Rietveld refinement^51^ was performed within the FullProf 3.5 suite^52^ using a Thompson-Cox-Hastings (TCH) pseudo-Voigt profile function^53,54^ and a polynomial background model. Isotropic vibration modes were assumed for all atoms. Additionally, a size-strain analysis was performed by the Rietveld method using a Highscore program^55^. For the purpose of the size-strain analysis, the Si powder (99.999%, Koch-Light Lab. Ltd., UK; spherical particles with diameter of 1 μm) was used as a standard for the instrumental diffraction line broadening. The crystallite size and the lattice strain in the same sample were determined simultaneously.

### Microstructural characterization

The transmission and scanning transmission electron microscopy (TEM and STEM, respectively) studies were carried out using a probe Cs-corrected Jeol ARM 200 CF microscope operated at 80 kV. Powder samples were suspended in the ethanol and dispersed on a lacey-carbon coated copper grid for the TEM analysis.

### Magnetization study

The magnetization *M* of pressed polycrystalline pellet was measured with a MPMS-5 magnetometer equipped with superconducting quantum interferometer device (SQUID). The pellet was fixed alone within plastic straw used for the measurements, without using the ampoule, grease, or any other medium, so that only a pure sample was measured and there was no need for any corrections. Also, the sample was not exposed to a contamination of any type of (para)magnetic impurities during the preparation for measurements, as well as during thermo-atmospheric treatments within the oven. The temperature dependence of magnetization, *M*(*T*), was measured in different magnetic fields in the temperature range of 2–400 K for initial characterization, and the field of 0.1 T was chosen as the most appropriate for extended investigation of the magnetization after thermal and atmospheric treatments. Additionally, the field dependences of magnetization, *M*(*H*), including magnetic hysteresis loops, were measured at several stable temperatures in the fields of up to 5 T. A furnace with quartz tube was installed near the magnetometer. The atmosphere within the tube was evacuated with a vacuum pump, flushed with Ar several times, and exposed thereafter to a slow Ar flow under pressure only slightly above the atmospheric pressure. Under these conditions the heating and cooling of the pellet were performed in pure Ar without presence of an air.

## Supplementary information

Supplementary Information for: Magnetic oxygen stored in quasi-1D form within BaAl2O4 lattice

## Data Availability

The authors declare that the data supporting the results of this study are available within the article and its Supplementary Information file. Further details of the crystal structure investigations may be obtained from the Fachinformationszentrum Karlsruhe, 76344 Eggenstein-Leopoldshafen (Germany), on quoting the CSD-434157 Depository Number.
