# Supplementary material for: Magnetic oxygen stored in quasi-1D form within BaAl2O4 lattice
Source: Sci Rep. 2019 Oct 22;9:15158. doi: 10.1038/s41598-019-51653-4 (PMC6805866; doi:10.1038/s41598-019-51653-4)
Supplement: Supplementary file 1 — Supplementary Information for: Magnetic oxygen stored in quasi-1D form within BaAl2O4 lattice [file 41598_2019_51653_MOESM1_ESM.docx]

**Supplementary Information for: Magnetic oxygen stored in quasi-1D form within BaAl_2_O_4_ lattice**

Martina Vrankić^1,*^, Ankica Šarić^1^, Sanja Bosnar^2^, Damir Pajić^3,*^, Jure Dragović^3^, Angela Altomare^4^, Aurelia Falcicchio^4^, Jasminka Popović^1^, Marijana Jurić^2^, Mladen Petravić^5^, Ivana Jelovica Badovinac^5^, Goran Dražić^6^

^1^Division for Materials Physics, Center of Excellence for Advanced Materials and Sensing Devices, Division for Materials Physics, Ruđer Bošković Institute, Bijenička 54, 10000 Zagreb, Croatia

^2^Division of Materials Chemistry, Ruđer Bošković Institute, Bijenička 54, 10000 Zagreb, Croatia

^3^Department of Physics, Faculty of Science, University of Zagreb, Bijenička 32, 10000 Zagreb, Croatia

^4^Institute of Crystallography-CNR, via Amendola 122/o, 70126 Bari, Italy

^5^Department of Physics and Center for Micro- and Nanosciences and Technologies, University of Rijeka, Radmile Matejčić 2, 51000 Rijeka, Croatia

^6^Department of Materials Chemistry, National Institute of Chemistry, Hajdrihova 19, Ljubljana, Slovenia


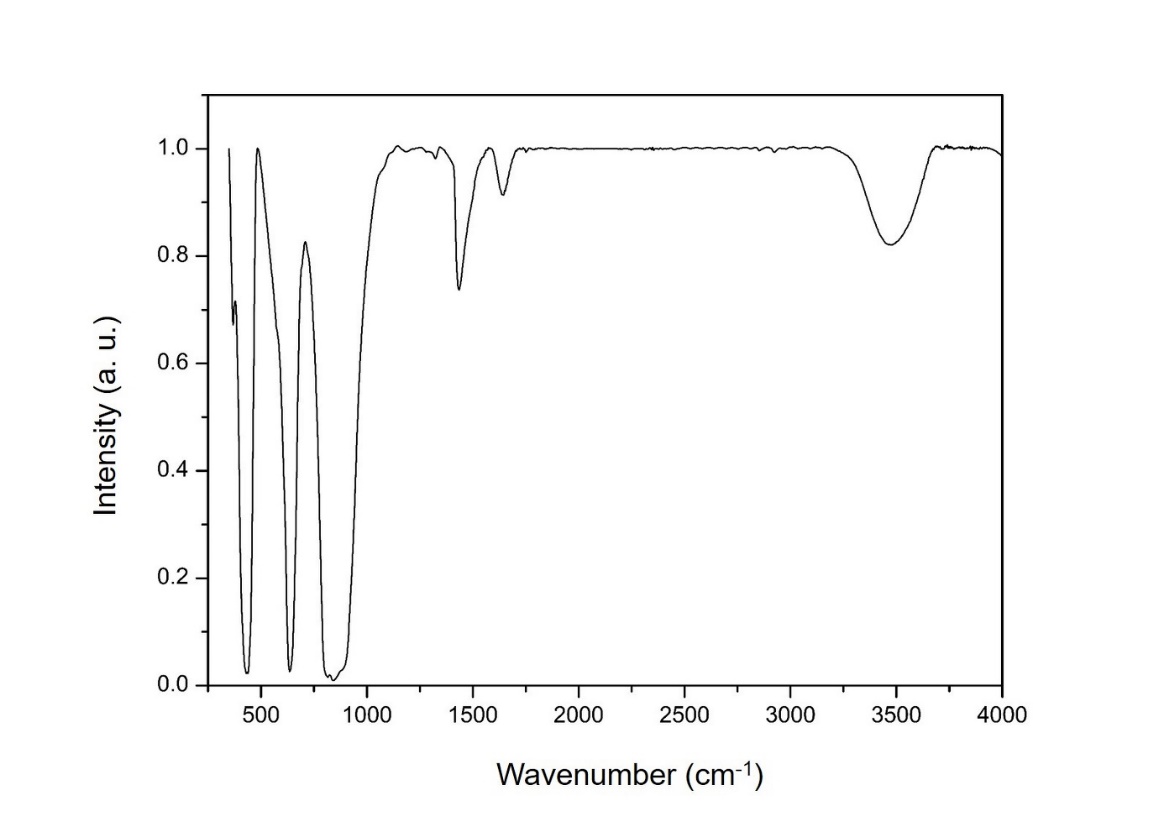


**Supplementary Figure 1.** The FTIR spectra of BaAl_2_O_4_ powder thermally treated at 1100 °C in the wavenumber ranging from 350 to 4000 cm^–1^. The broad band centred around 3480 cm^−1^, and one located at 1640 cm^–1^ originate from the stretching mode of of hydroxyl group and the deformation vibration of H–O–H typical for the water molecules absorbed by the sample, respectively^38,39^. The stretching band at ~1435 cm^-1^ is assigned to carbon, that exists as a carbonate in BaAl_2_O_4_^31,40,41^. The CO_2_ could be driven off by heating the BaAl_2_O_4_ samples overnight at high temperature (Supplemetary Fig. 2). Absorbed peaks centred at 430, 630 and 830 cm^–1^ are typical for the BaAl_2_O_4_ species^42^.

**
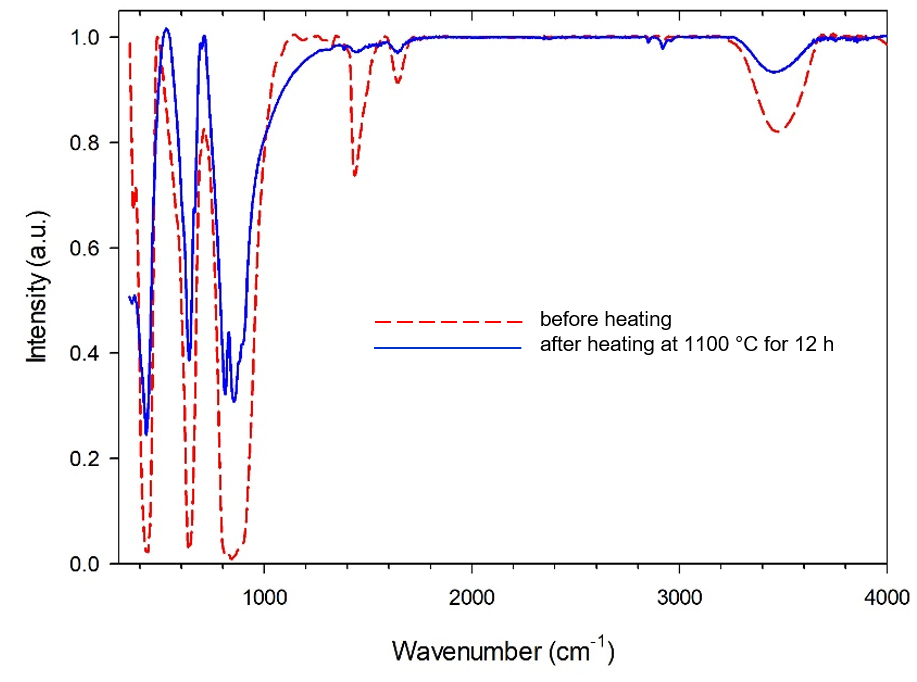
**

**Supplementary Figure 2.** The FTIR spectra of the BaAl_2_O_4_ after staying in the air (dashed red line) and immediately after heating treatment at 1100 °C for 12 h (solid blue line). The intensity of typical carbonate absorption band at ~1435 cm^-1^ significantly decreases after the heat treatment at 1100 °C overnight.

Thermal stability characteristics and oxygen intake properties were evaluated by means of thermogravimetric (TG) experiment carried out upon heating in controlled atmosphere (i.e. pure O_2_).


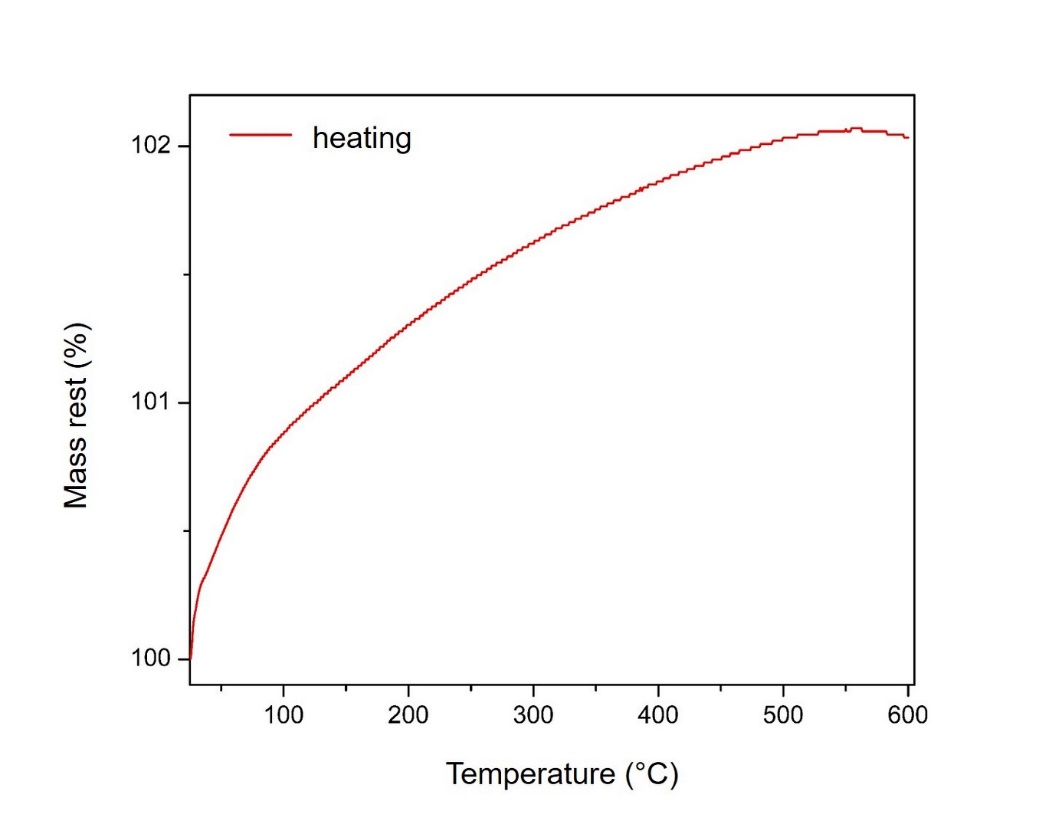


**Supplementary Figure 3.** The TGA curve of a BaAl_2_O_4_ sample accomplished in second cycle of heating in pure O_2_ from the RT to 600 °C with a scan rate of 2 °C/min. The TG experiment performed in pure O_2_ shows the significant weight gain of 2.034 % and complement a tendency of BaAl_2_O_4_ to intake the oxygen in the wide temperature range, from RT up to 600 °C.

**Supplementary Table 1.** Summary of structure refinements for the BaAl_2_O_4_ obtained from the Rietveld analysis of a high quality laboratory XRPD data collected at RT (λ=1.54056Å). Estimated errors in the last digits are given in the parentheses.

| **Global parameters** | **Structural and microstructural parameters** | | | | | | | |
| --- | --- | --- | --- | --- | --- | --- | --- | --- |
| profile function:  pseudo Voigt  asymmetry type:  Finger, Cox, Jephcoat  R_profile_ (%):  4.45  R_weighted profile_ (%):  5.94  formula sum:  Ba_2.00_Al_4.00_O_8.00_  formula mass (g/mol):  510.617  density calculated (g/cm^3^): 4.086  space group (No.):  *P*6_3_22 (182)  V (10^6^ pm^3^):  207.510(5) | *a* (Å): 5.22111(6)  *b* (Å): 5.22111(6)  *c* (Å): 8.78981(14)  alpha = beta (°): 90  gamma (°): 120 | | | | | | | |
|  | Atom | Wyck.  position | | Coordinates | | Occupancy | | U_iso_ (Å^2^) |
|  | Ba1  Al1  O2  O3 | *2b*  *4f*  *6g*  *2d* | | 0.00000  1.00000  0.75000  0.33330  0.66670  0.5551(6)  0.00000  0.637(2)  0.50000  0.33330  0.66670  0.75000 | | 0.16667  0.33333  0.50000  0.16667 | | 0.016(2)  0.022(2)  0.33(3)  0.39(1) |
|  | interatomic distances M-O (Å) | | | | | | | |
|  | Ba1-O1 x 6  Ba1-O2 x 3 | | 2.902(4)  3.0144(1) | | Al-O1 x 1  Al-O1 x 1 Al-O1 x 1  Al-O2 x 1 | | 1.737(5)  1.737(6)  1.737(8)  1.713(6) | |
|  | crystallite size (nm): 63.7(1)  lattice strain (%): 0.01(1) | | | | | | | |

**Supplementary Information References**

31. Zhang, L.-W., Wang, L. & Zhu, Y-F. Synthesis and performance of BaAl_2_O_4_ with a

wide spectral range of optical absorption. *Adv. Funct. Mater.* **17,** 3781–3790

(2007).

38. Staszak, W., Zawadzki, M. & Okal, J. Solvothermal synthesis and

characterization of nanosized zinc aluminate spinel used in iso-butane

combustion. *Alloys. Comp.* **492,** 500–507 (2010).

39. Zhu, Z., Liu, F., & Zhang, W. Fabricate and characterization of Ag/BaAl_2_O_4_ and its

photocatalytic performance towards oxidation of gaseous toluene studied by FTIR

spectroscopy, *Mater. Res. Bull.* **64,** 68–75 (2015).

40. Rodehorst, U., Carpenter, M. A., Marion, S., Henderson & M. B. Structural phase

transitions and mixing behaviour of the Ba-aluminate (BaAl_2_O_4_)-Sr-aluminate

(SrAl_2_O_4_) solid solution, *Mineral. Mag.* **67(5)**, 989–1013 (2003).

41. Kochiyil Cherikkallinmel, S., et al., Sodium aluminate from waste aluminium

source as catalyst for the transesterification of Jatropha oil, *RSC Adv.* **5,** 46290–

46294 (2015).

42. Luan, C. N., Ma, J., Zhu, Z., Kong, L. Y. & Yu, Q. Q. Sol-gel synthesis and

characterization of BaAl_2_O_4_ and Co:BaAl_2_O_4_ nanoparticles. *Adv. Mater. Res*.

**148/149,** 1067–1070 (2011).
